# Glycyrrhiza polysaccharides may have an antitumor effect in γδT cells through gut microbiota and TLRs/NF‐κB pathway in mice

**DOI:** 10.1002/2211-5463.13800

**Published:** 2024-04-11

**Authors:** Yinxiao Chen, Zhaodong Li, Liding Bai, Bin Lu, Yanfei Peng, Pengjuan Xu, Xinbo Song, Yuhong Bian, Xiangling Wang, Shuwu Zhao

**Affiliations:** ^1^ College of Integrative Medicine Tianjin University of Traditional Chinese Medicine China

**Keywords:** glycyrrhiza polysaccharides, gut microbiome, gut mucosal immunity, NF‐κB pathway, γδT cells

## Abstract

Tumor immunotherapy can be a suitable cancer treatment option in certain instances. Here we investigated the potential immunomodulatory effect of oral glycyrrhiza polysaccharides (GCP) on the antitumor function of γδT cells in intestinal epithelial cells in mice. We found that GCP can inhibit tumor growth and was involved in the regulation of systemic immunosuppression. GCP administration also promoted the differentiation of gut epithelia γδT cells into IFN‐γ‐producing subtype through regulation of local cytokines in gut mucosa. GCP administration increased local cytokine levels through gut microbiota and the gut mucosa Toll‐like receptors / nuclear factor kappa‐B pathway. Taken together, our results suggest that GCP might be a suitable candidate for tumor immunotherapy, although further clinical research, including clinical trials, are required to validate these results.

## Graphical abstract

*In vivo* experiments in mice suggest that *Glycyrrhiza uralensis* polysaccharides (GCP) from *G. uralensis* Fisch can increase local cytokine levels in the intestinal mucosa through gut microbiota and the intestinal mucosal Toll‐like receptors / nuclear factor kappa‐B pathway. GCP administration also promotes differentiation of intestinal epithelial γδT cells into IFN‐γ producing subtypes, which inhibit tumor growth and regulate systemic immunosuppression in mice.

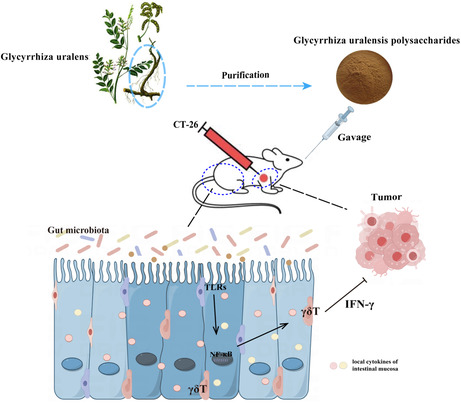

**Abbreviations**
CD3: cluster of differentiation 3
CD4: cluster of differentiation 4
CD8: cluster of differentiation 8
CT‐26: colon tumor cell line 26
DAB: 3,3*N*‐diaminobenzidine tertrahydrochloride
DAPI: 4′,6‐diamidino‐2‐phenylindole
GCP: *Glycyrrhiza uralensis* polysaccharides
IFN‐γ: interferon‐γ
NKG2D: natural killer group 2 member D
RT‐PCR: reverse transcription polymerasechain reaction
SPF: specific pathogen free
SPSS: Statistics Package for Social Science
TCR: T cell receptor
TLR4: toll‐like receptor 4

The incidence of cancer is on the rise worldwide, which is one of the major diseases threatening human life and health [1, 2]. Although traditional treatments such as surgical resection, radiotherapy, and chemotherapy are effective against malignant tumors, they often result in serious side effects [3]. As a result, tumor immunotherapy has emerged as a potent treatment option, encompassing techniques like cellular permeation immunotherapy and cytokine therapy [4]. Moreover, there is an urgent need to identify new drug candidates, enhance the organic immunity to improve the efficacy of chemotherapy and prevent metastasis in a variety of pathways, and regulate the tumor immune microenvironment to boost tumor immunotherapy and chemotherapy. Polysaccharides have various biological functions [5, 6], some of which can produce immunomodulatory activity by modulating the immune response in the intestinal mucosa [7, 8]. This includes actions on immune cells such as T cells, B cells, and macrophages. γδT cells have an affinity for developing tumors and have been shown to play a role in tumor combat and immune surveillance [9]. *Glycyrrhiza uralensis* polysaccharides (GCP) are one of the main active components of *G. uralensis* Fisch (also named Licorice in English) and play an important role in antiinflammation, immunomodulation, antioxidation, and gut microbiota regulation [10], especially in antitumor immunity [10, 11]. Studies have shown that various herbal polysaccharides can inhibit tumor growth through immunomodulation [12]. GCP can promote the maturation of dendritic cells and enhance their immunomodulatory functions, as well as promote the release of interleukin‐1 (IL‐1), IL‐6, and IL‐12 from macrophages [13]. In our previous studies, a series of stable methods for the extraction, isolation, and purification of GCP have been established [14, 15]. By this method, we derived low molecular components of GCP fractions and confirmed that the safe dose of GCP for experiments is 500 mg·kg^−1^, further confirming its immunomodulatory and antitumor potential [10, 16, 17]. Also, we further explored the possible mechanisms of the GCP regulating gut epithelia γδT cells to antitumor in this study.

The gut mucosal immune system is the starting point for oral administration of polysaccharides to exert immune efficacy and regulate human immune function. The human gut is colonized by more than 100 trillion microbes and is closely related to intestinal mucosal immunity [18, 19]. Despite interindividual variations, according to metagenomic studies intestinal bacteria mainly contains *Firmicutes*, *Bacteroidetes*, *Actinobacteria*, *Proteobacteria* (dominant part), *Verrucomicrobia*, *Deferribacteraceae*, and *Euryarchaeota* (uncommon component). Through a variety of intricate biological processes, an imbalance in the gut microbiota can impact intestinal immunity and contribute to the emergence and progression of several illnesses, including tumors [20]. Research has indicated that polysaccharides may serve as viable prebiotics to modulate gut microbial diversity and composition [21]. *Tremella fuciformis* polysaccharides, for instance, have the potential to greatly boost the diversity of the gut microbiota, restore the relative abundance of *Lactobacillus*, *Odoribacter*, *Helicobacter*, *Ruminoccaceae*, and *Marinifilaceae*, and augment antiinflammatory cytokines, eventually inducing Foxp3^+^ T cell expansion [22]. According to the latest research, *Cordyceps sinensis* polysaccharides could reverse Cy‐induced gut microbial dysbiosis by modulating the microbial community structure and composition. It could also regulate T helper (Th) cell differentiation by stimulating cytokine secretion and transcription factor production, thus restoring Cy‐induced immunosuppression in the intestinal mucosa [23]. Our earlier research has shown that the gut microbiota plays a critical role in the antitumor actions of GCP [10]. However, it remains to be further confirmed whether the antitumor effects of GCP on intestinal epithelial γδT cells are related to alteration in intestinal flora.

As the key components of the innate immune system, Toll‐like receptors (TLRs) are expressed in intestinal epithelial cells and mucosal immune cells and are activated by cytokines induced by the nuclear factor kappa‐B (NF‐κB) pathway [24, 25]. To prevent excessive immune response‐induced intestinal microbiota, the intestinal epithelium expresses few TLRs in healthy settings. Consequently, TLRs are crucial for preserving the intestinal mucosa's and even the organism's immunological homeostasis. TLRs can rapidly detect and activate in response to a local lesion in the intestinal mucosa or alteration in the intestinal microbiota brought on by the body's lesion, thereby regulating intestinal mucosal immune cells, enhancing local immunity, and influencing the release of associated cytokines [26]. Based on these, we mainly study the immunomodulatory effect of oral GCP on the antitumor function of γδT cells in intestinal epithelial cells. In turn, it provides new ideas and approaches for GCP antitumor immunity.

## Materials and methods

### Drug preparation

The extraction and purification of GCP were conducted as described below. Briefly, GCP was prepared by water extraction and alcohol precipitation. The *G. uralensis* crude polysaccharide was prepared in a 5% aqueous solution. The water‐insoluble substances were removed by centrifugation at 6000 ***g*** for 10 min. After the supernatant was concentrated, alcohol precipitation was carried out with a 57–82% ethanol concentration. The precipitate was washed several times with 95% ethanol and acetone in turn. Then the GCP was deproteinized using the Sevag method. After dialysis, the polysaccharide solution was freeze‐dried and GCP was therefore obtained. GCP with an average molecular weight of 5.5 × 10^4^ Da [14], and preserved in the Tianjin University of Traditional Chinese Medicine Institute of Traditional Chinese Medicine until use.

### Cell culture and reagent

Mouse colon cancer cell line CT‐26 (TCM37) was purchased from the Institute of Biochemistry and Cell Biology, Chinese Academy of Sciences (Shanghai, China). The cell line was cultured in PRMI‐1640 medium (Solarbio, Beijing, China) supplemented with 10% fetal bovine serum (FBS; Gibco, Grand Island, NE, USA) in a 37 °C incubator.

### Animals and experimental design

The WT male BALB/c mice (6 weeks old, 21–23 g, SCXK2021‐0006) were purchased from Beijing Vital River Laboratory Animal Technology. The above‐mentioned mice were fed in the Experimental Animal Center of Tianjin University of Traditional Chinese Medicine (SYXK(Jin)2020‐0005). Animal experiments were carried out in strict accordance with the regulations of the Experimental Animal Ethics Committee of Tianjin University of Traditional Chinese Medicine (TCM‐LAEC2020074). During the study, the experimental mice were free to drink water and eat freely and were kept at room temperature (22 ± 2) °C and humidity of 40 ± 10%. The physical condition of the mice was monitored every day. No mice became severely ill or died at any time before the experimental endpoint. To establish a tumor model, 4 × 10^5^ C T‐26 cells were subcutaneously injected into the axilla of BALB/c mice. Mice were randomly divided into two groups on day 3 after tumor cell inoculation, including the model group (Model) and the GCP treatment group (GCP). A control group (Control) was also established. The GCP group was given orally a dosage of 500 mg·kg^−1^ GCP once a day, which was dissolved in autoclaved water and administered continuously for 21 days. The model groups were given phosphate‐buffered saline (PBS) in the same way, with a dosage of 0.2 mL per animal. Twenty‐one days after administration, mice were anesthetized with 10% chloral hydrate injected intraperitoneally (0.5 mL·kg^−1^). After blood collection, mice were quickly killed by dislocating the cervical region.

### Weight measurement of mice

The body weight of each group of mice was regularly weighed every day. Its change was calculated based on the body weight of the mice on the first day.

### General condition of the mice

During GCP administration, the life quality of mice was observed, including water intake and food intake, and the mental state was recorded daily. After 21 days of administration, the mice were sacrificed and the spleen was removed. The formula for calculating the spleen index is spleen weight (mg)/body weight (g).

### Tumor volume calculation and weight measurement

The time of tumor formation was recorded as the time of palpable tumor. During GCP administration, the tumor volume of mice was observed and measured daily. The tumor volume of mice was calculated by the formula: 1/2 long diameter × short diameter^2^. Twenty‐one days after dosing, mice were sacrificed, tumors were removed, and the mass was recorded.

### Hematoxylin–eosin staining

Tumor tissue was placed in 4% paraformaldehyde (PFA) for 24–48 h. The fixed tissues embedded in paraffin were sliced into 5‐μm‐thick sections. The tissue sections were mounted on glass slides and stained with hematoxylin and eosin (HE). Samples were captured by microscopy.

### Immunohistochemistry and immunofluorescence

Mice tumor and spleen samples were fixed in 4% PFA for 24–48 h, embedded in paraffin, and mounted on slides (5‐m coronal sections). The tissue slices were dewaxed twice in xylene for 10 and 5 min, and dehydrated in a gradient ethanol solution (100%, 95%, 80%, and 70%). Slices were rinsed three times and 2 min each with PBS. Antigen retrieval was performed in citrate antigen retrieval solution (Solarbio) at 95 °C for 10 min in a pressure cooker. After cooling naturally in the retrieval solution, slices were rinsed with PBS, followed by blocking with 5% bovine serum albumin at room temperature for 1 h, and the excess liquid was shaken off. The primary antibody information utilized is as follows: Ki‐67 antibody and anti‐CD3 antibody (Proteintech, Wuhan, China; dilution: 1:1500) overnight at 4 °C. After rinsing with PBS, the biotinylated secondary antibody was initially applied for 30 min, after reaction enhancer in Universal Two‐step Detection Kit (Proteintech) was used for an additional 30 min. 3,3′‐Diaminobenzidine tetrahydrochloride hydrate was utilized for detection. Slices were then counterstained with hematoxylin to stain the nuclei. Samples were captured by microscopy. For immunofluorescence, FITC labeled fluorescent primary antibody γδT (Santa Cruz Bio, Santa Cruz, CA, USA; dilution: 1:500) and CD27 (R&D Systems, Minneapolis, MN, USA; dilution: 1:3000), were incubated overnight at 4 °C. On the next day, sections were rewarmed, washed with PBS, and Alexa Fluor 647‐labeled fluorescent secondary antibody (ABX BiO, Beijing, China; dilution: 1:400) was incubated in the dark for 1 h. After staining with DAPI, the slices were observed by fluorescence microscope and photographed.

### Flow cytometry analysis of gut mucosal lymphocyte subsets

The cleaned gut was cut into 3‐cm pieces and added to the lysate (which contained HBSS with 10 mm HEPES, 8% FBS, 4 mm EDTA, and 0.5 mm DTT; Solarbio). The lysates were placed on a shaking incubator at 250 r.p.m. for 37 °C for 20 min. This process was repeated three times to collect the cell lysates. The lysate was filtered by a 40‐μm filter and separated by Percoll gradient centrifugation. The intermediate milky cell layer was collected and washed twice with precooled PBS. The resulting cell suspension was conducted for surface staining and intracellular‐stained for flow cytometry. For surface staining, 1 × 10^6^ cells were added with fluorescent‐labeled antibodies (CD3‐APC, CD4‐FITC, CD8‐PE, TCRγ/δ‐FITC, NKG2D‐PE, and CD27‐PE; BioLegend, San Diego, CA, USA), and incubated at 4 °C for 30 min under dark conditions. The cells were washed with PBS and resuspended with 1 mL PBS under dark conditions. For intracellular‐staining, PMA Phorbol 12‐myristate 13‐acetate 0.5 ng·mL^−1^ (Yeasen Biotechnology, Shanghai, China), ionomycin 1 ng·mL^−1^ (Solarbio), BFA Brefeldin A 5 ng·mL^−1^ (Yeasen Biotechnology) to the centrifuge tube, and incubated in a cell incubator for 6 h. To this was added 1 mL of cell fixative solution and incubated at 4 °C for 10 min, then 0.5 mL added cell permeabilization solution and incubated at 4 °C for 10 min. The IFNγ‐PE antibody was used for staining. Flow cytometry was used for cell population detection and the analyzed data were performed using guava software [27].

### Fecal sample collection and 16S rRNA amplicon sequencing

The feces in the intestinal tract of the mice were collected and stored in a 1.5‐mL sterile centrifuge tube. Fecal samples were transported on dry ice, and APTBIO was entrusted to perform 16S rDNA amplicon sequencing analysis.

### RNA extraction and real‐time PCR analysis (RT‐qPCR)

The total RNA of the gut epithelial tissue was extracted using an RNA Fast kit (Yeasen Biotechnology). The cDNA synthesis was performed by the HiScript®II Reverse Kit (TIANGEN, Beijing, China). The RT‐qPCR was performed using SuperReal SYBR Green PCR Mix (TIANGEN) on the IQ5 PCR System (Biorad, Hercules, CA, USA). The reaction procedures were: 94 °C for 1 min, 94 °C for 20 s, 58 °C for 30 s, 72 °C for 30 s, with 40 cycles. The primers used are listed in Table 1. The relative expression of target genes was calculated with $2^{-\Delta \Delta C_{t}}$. GAPDH was used as the internal control for RT‐qPCR.

**Table 1:** The primers are used for RT‐qPCR

Gene name	Primer	Sequence (5′–3′)
*TLR4*	Forward	5′CCCTGCCACCATTTACAGTT3′
	Reverse	5′ATCAGAGTCCCAGCCAGATG3′
*TLR2*	Forward	5′TCCAGGCCAAGAGGAAG3′
	Reverse	5′ATGAGGTTCTCCACCCAAT3′
*TLR6*	Forward	5′CTCATCTTGCTGGAACCC3′
	Reverse	5′CACGTTTGCCCTTCTCA3′
*TLR9*	Forward	5′CAGGAGCGGTGAAGGTG3′
	Reverse	5′GAAGGGAGGTCAGATTGGC3′
*GADPH*	Forward	5′GGCACAGTCAAGGCTGAGAA3′
	Reverse	5′ATGGTGGTGAAGACGCCAGTA3′

### Western blotting

The gut mucosal tissues were scraped and resuspended in 0.5 mL RIPA buffer (CWBIO, Taizhou, China). Tissue lysates were placed on ice for 1 h. After centrifugation at 10,000 ***g*** for 20 min, the protein concentration was determined using the BCA kit (CWBIO). Twenty microgram samples were electrophoresed via 8% SDS/PAGE (Yeasen Biotechnology) and transferred onto a PVDF membrane. Samples were blocked with 5% nonfat milk in TBST solution at room temperature for 2 h and then probed with NF‐κB p65, IκB‐α, p‐IκB‐α (Abclonal Technology, Wuhan, China; dilution: 1:1000), β‐actin (Abclonal Technology; dilution: 1:10 000) at 4 °C overnight. Secondary antibodies used for detection included HRP‐conjugated antirabbit IgG (Abclonal Technology; dilution: 1:10 000), and the protein expression was detected using an ECL chemiluminescence detection kit (Boster, Wuhan, China).

### ELISA assay

The mice serum samples and gut mucosal homogenate were collected. IL‐7, IFN‐γ, IL‐12, and TNF‐α of serum and IL‐7, TNF‐α, IL‐2, IL‐12, and IL‐21 of gut mucosa were measured by ELISA. The ELISA procedure followed the instructions of the ELISA Kit (Multi Sciences, Hangzhou, China).

### Statistical analysis

All data analysis was performed using spss 17.0 (Chicago, IL, USA). The data were presented as means ± standard error of the mean (SEM) for each individual experiment. The *t*‐test was used for comparison between the two groups, and a one‐way ANOVA analysis of variance was used among the multiple groups. *P* < 0.05 was considered statistically significant. Images shown herein were all produced by graphpad prism 8.0（Santiago, MN, USA software.

## Results

### GCP exhibits antitumor activity in tumor‐bearing mice

**Fig. 1:**
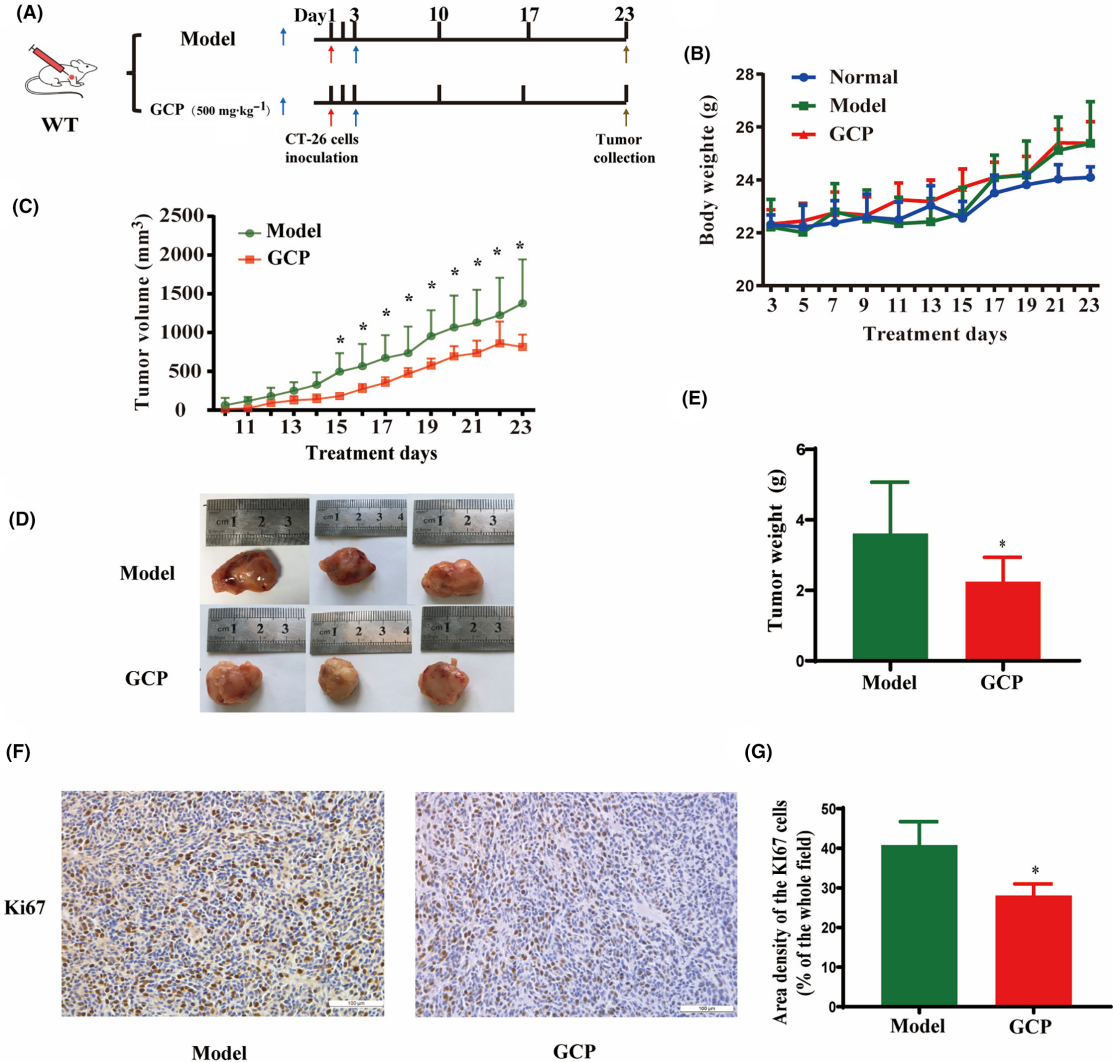
GCP inhibited the development of tumors *in vivo*. (A) Diagram of the *in vivo* experimental timeline. (B) Body weight. (C) Tumor growth curves (*P < 0.05, *n* = 6 Model :vs. GCP). (D) Representative images of stripped subcutaneous tumors. (E) Tumor weight (*P < 0.05, *n* = 6 Model :vs. GCP). (F) Representative images of Ki‐67 immunohistochemical staining of tumors. (G) Immunohistochemical staining statistical results for Ki‐67 in tumors (*P < 0.05, *n* = 6 Model :vs. GCP).

To assess the effect of GCP on antitumor activity *in vivo*, BALB/c mice were used to establish subcutaneous colon cancer tumors with CT‐26 cells, and GCP was orally administered for 21 days at the 3rd day after tumor cell inoculation (Fig. 1A). As depicted in Fig. 1B, treated with GCP (500 mg·kg^−1^) did not affect the body weight of tumor‐bearing mice compared with the Model group. During the 21‐day treatment period, the tumor volume of the Model group showed a significant increase after 14 days, while the tumor volume of the GCP‐treated group alleviated this trend (Fig. 1C). After 21 days of GCP treatment, mice were sacrificed on the 23rd day, and tumor weight and volume were compared between the GCP‐treated group and the Model group. Compared with the Model group, the tumor weight and size of the GCP‐treated group were significantly reduced (Fig. 1D,E) (*P* < 0.05). Histological examinations of the tumor tissues showed that the Model group structure was clear, with dense nuclei, while the cell structure was not so tight in the GCP‐treated group (Fig. S1a). Immunohistochemistry analysis of tumors showed fewer Ki67‐positive cells in the GCP‐treated group than in the Model group (Fig. 1F,G) (*P* < 0.05). These results indicated that GCP could efficiently suppress tumor growth.

### GCP enhanced antitumor immunity in tumor‐bearing mice

**Fig. 2:**
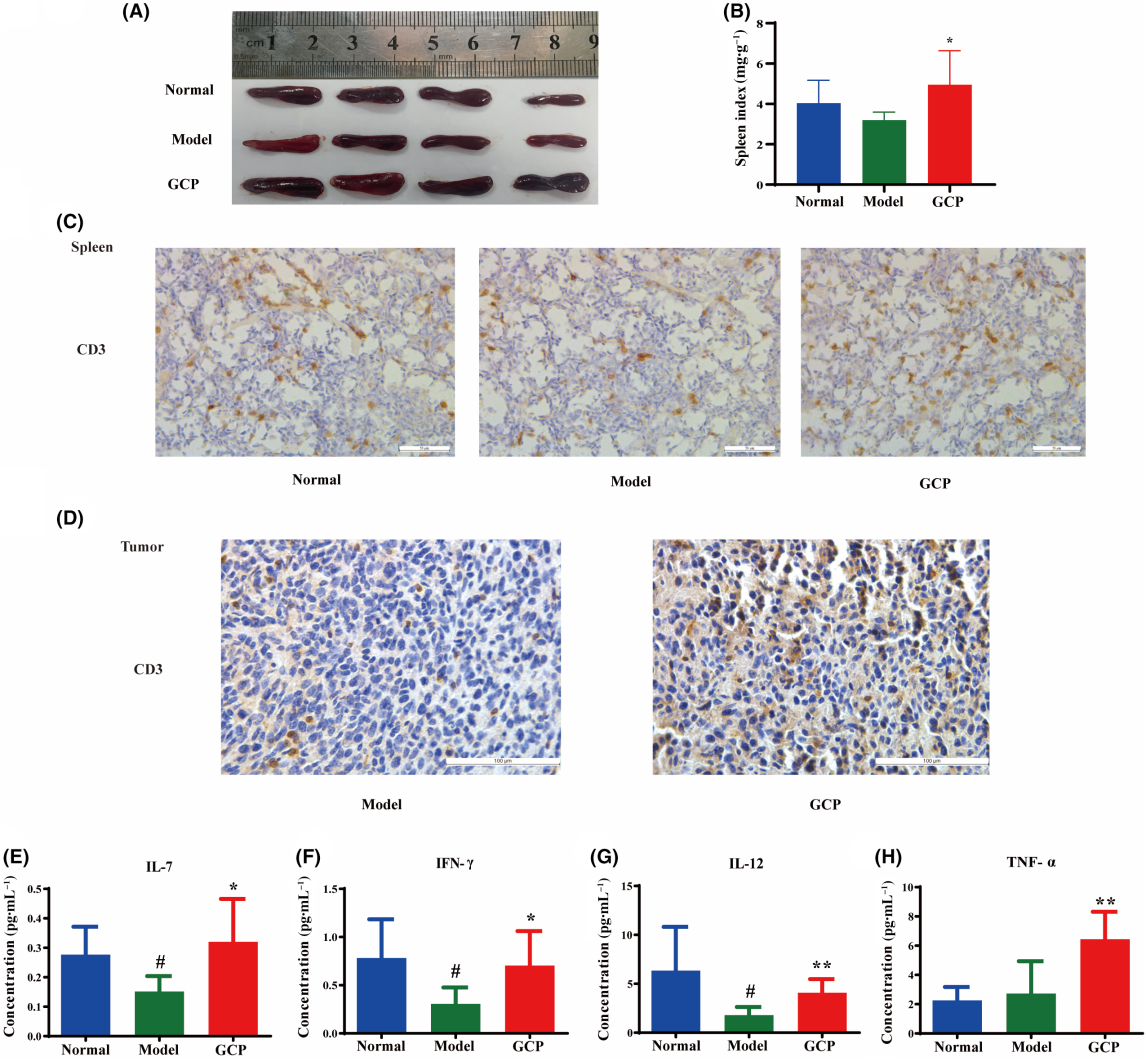
GCP enhanced antitumor immunity *in vivo*. (A) Representative images of the spleen dissected from the tumor‐bearing model. (B) Spleen index (*P < 0.05, *n* = 6 Model :vs. GCP). (C) Representative images of CD3 immunohistochemical staining of spleens (Scale bar, 50 μm). (D) Representative images of CD3 immunohistochemical staining of tumors (Scale bar, 100 μm). (E–H) ELISA was used to detect cytokine IL‐7, IFN‐γ, IL‐12, TNF‐α concentration in blood serum (**P < 0.01, *n* = 6 Model :vs. GCP *P < 0.05 Model :vs. GCP ^#^ *P* < 0.05 Normal :vs. Model).

The organ index results showed that the spleen index in the GCP‐treated group increased significantly compared to the Model group (*P* < 0.05) (Fig. 2A,B). To detect whether the antitumor effect of GCP was associated with immunological enhancement in tumor‐bearing mice, we next analyzed the proportion of CD3^+^ T cells as well as the expression level of cytokines from mice. Anti‐CD3 antibody staining results in the spleen showed more T cells in the GCP‐treated group than the Model group (Fig. 2C). We also examined the effect of GCP on immune cell infiltration in tumor tissue, and there was a significant increase of CD3^+^ T cells in the GCP‐treated group compared to the Model group (Fig. 2D). Compared with the Normal group (normal mice without tumor cell inoculation), the expression level of antiinflammatory factors IL‐7, IFN‐γ, and IL‐12 in the Model group were significantly reduced (*P* < 0.05), and the GCP‐treated group reversed this trend. And the GCP‐treated group could significantly increase the expression level of serum factor TNF‐α (*P* < 0.05) (Fig. 2E–H).

### GCP promoted the activation of γδT cells in gut epithelia and tumors

**Fig. 3:**
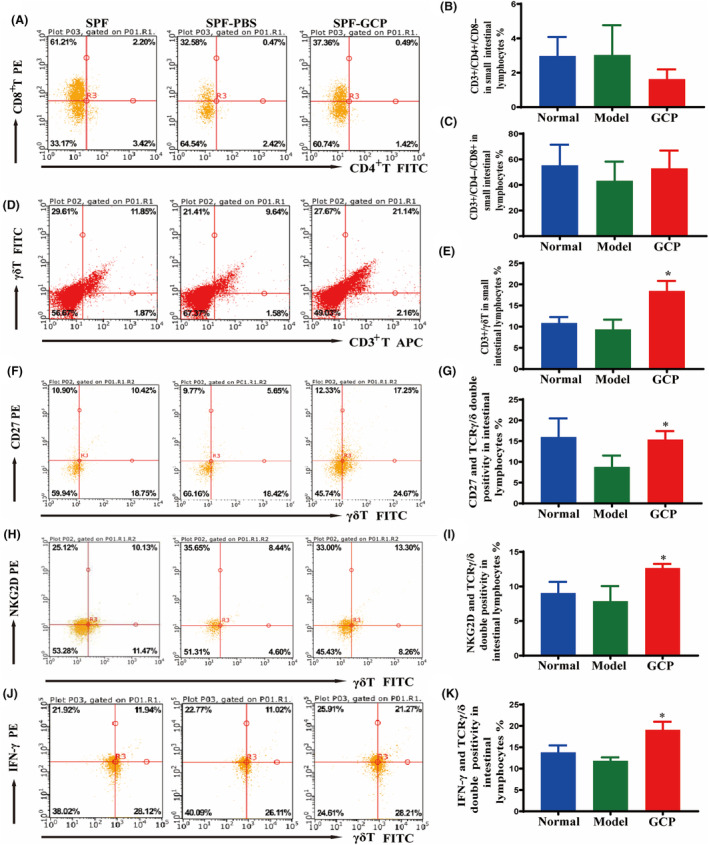
GCP helps to promote the proliferation and activated phenotypic differentiation of intestinal γδT cells. (A–C) Representative flow cytometry plots and statistics showed the ratio of lymphocyte subsets of intestinal CD3^+^/CD4^+^/CD8^−^T, CD3^+^/CD4^−^/CD8^+^T cells. (D,E) Representative flow cytometry plots and statistics showed the percentage of the intestinal CD3^+^/γδT cells (*P < 0.05 (*n* = 6) Model :vs. GCP). (F,G) Representative flow cytometry plots and statistics showed the activated phenotypes of CD27 in intestinal γδT cells (*P < 0.05 (*n* = 6) Model :vs. GCP). (H, I) Representative flow cytometry plots and statistics showed the activated phenotypes of NKG2D in intestinal γδT cells (*P < 0.05 (*n* = 6) Model :vs. GCP). (J,K) Representative flow cytometry plots showed the IFN‐γ production in intestinal γδT cells (*P < 0.05 (*n* = 6) Model :vs. GCP).

Our previous *in vitro* studies have found that GCP can promote the secretion of IL‐7 from intestinal epithelial cells, and promote the proliferation of T cells [16, 17]. Next, we detected CD3^+^T (CD3), CD4^+^T (CD4), CD8^+^T (CD8), and γδT (TCR‐γ/δ) surface markers by flow cytometry to further determine the role of GCP in T cells. The results showed that the proportions of CD3^+^/CD4^+/^CD8^−^T and CD3^+^/CD4^−^/CD8^+^T cells had no significant difference with the treatment of GCP (*P* > 0.05) (Fig. 3A–C). Compared with the Model group, the GCP‐treated group significantly increased the proportion of CD3^+^/γδT cells (*P* < 0.05) (Fig. 3D,E). These results suggest that GCP can significantly affect the proportion of γδT cells and may act as a potential antitumor immune cell.

To define the function of GCP on γδT cell activation, we analyzed γδT cell properties. By flow cytometry analysis, compared with the Model group, the proportion of activated (NKG2D^high^, CD27^high^) γδT cell proportion was significantly increased after administration of GCP (*P* < 0.05) (Fig. 3F–I), which suggested a positive role for GCP in gut epithelia γδT cell activation *in vivo*. Next, we assessed the expression of IFN‐γ between the Model group and the GCP‐treated group. By intracellular cytokine staining of gut epithelia γδT cells, we found that GCP triggered a significant increase in IFN‐γ production in γδT cells (*P* < 0.05) (Fig. 3J,K). Collectively, these findings revealed that GCP was a positive regulator of gut epithelia γδT cell activation and IFN‐γ production.

**Fig. 4:**
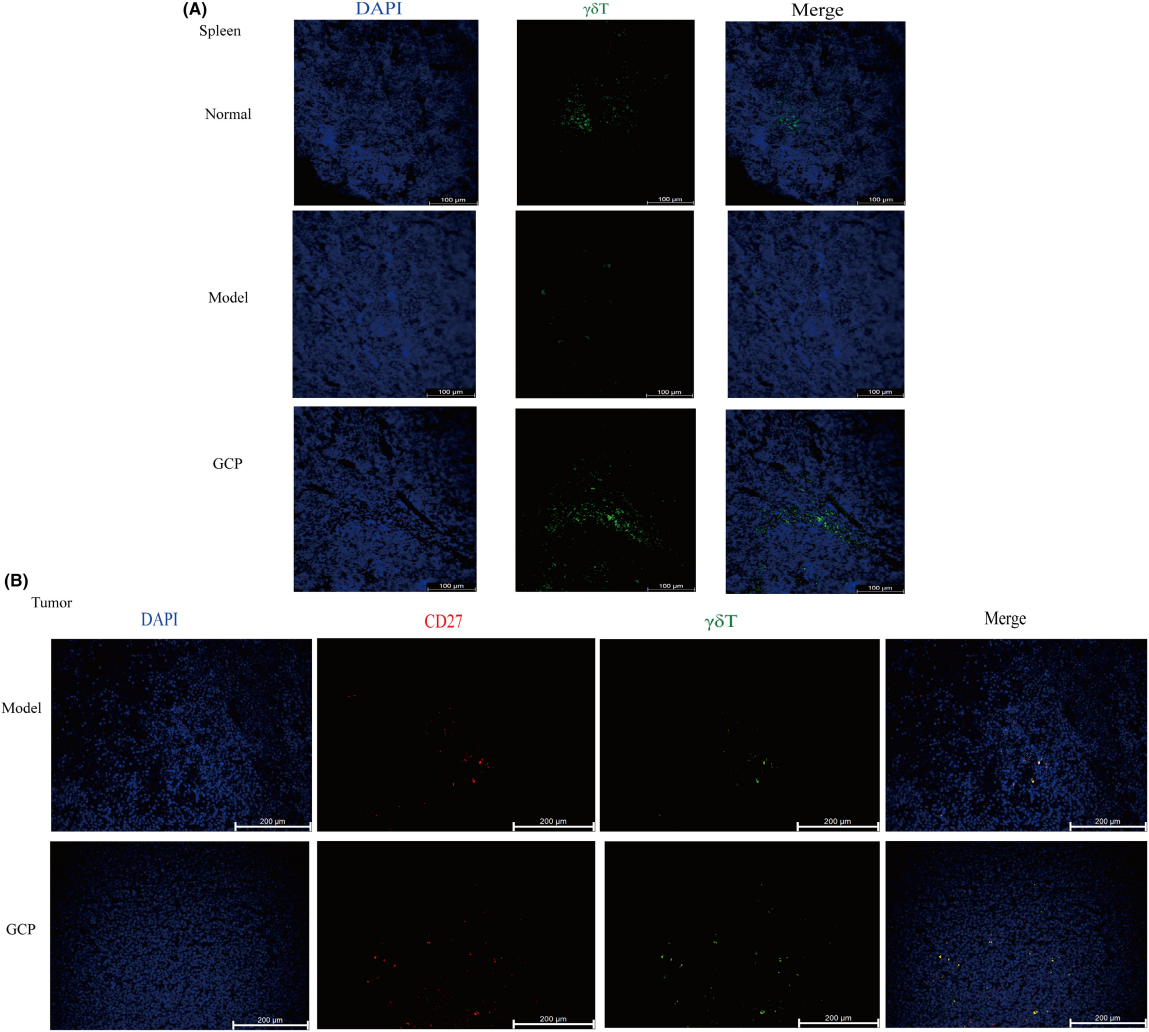
GCP can significantly increase the percentage of infiltration γδT cells in the spleen and tumors. (A) Representative images of γδT immunofluorescence staining of spleens (Scale bar, 100 μm). (B) Representative images of TCR‐γδ and CD27 immunofluorescence staining of tumors (Scale bar, 200 μm).

In addition, we also found that the anti‐TCR‐γδ antibodies staining in spleen sections showed more positive cells in the GCP‐treated group (Fig. 4A), and the number of tumor‐infiltrated CD27^+^γδT cells increased significantly in the GCP‐treated group compared to the Model group (Fig. 4B, Fig. S1b). The results indicated a vital role for γδT cells activated by GCP in tumor inhibition.

### GCP modulated the composition of gut microbiota in tumor‐bearing mice

**Fig. 5:**
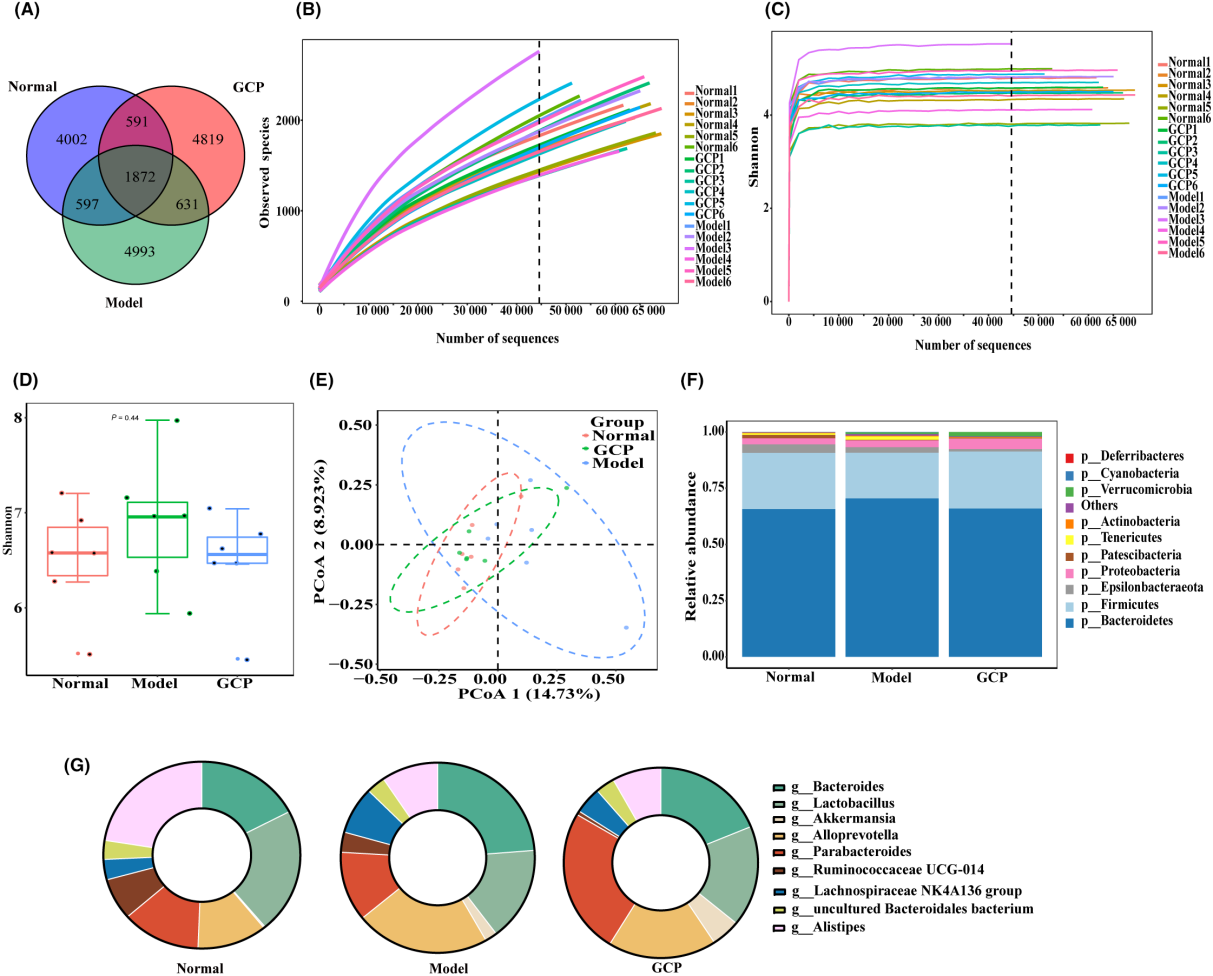
GCP restored the composition of gut microbiota in tumor‐bearing mice. (A) Venn diagram of the observed OTUs in Normal, Model, and GCP groups. (B) Curves of difference between Normal, Model, and GCP groups of rarefaction curves. (C) Curves of difference between Normal, Model, and GCP groups of Shannon curves. (D) Boxplot of difference between Normal, Model, and GCP groups of Shannon index. (E) Principal coordinate analysis (PCoA) of the microbiota based on the unweighted UniFrac distance metrics for the Normal, Model, and GCP groups. (F) The relative abundance of the three groups at the division level (Top 10). (G) The relative abundance of the three groups at the genus level (Top 10).

As our previous study indicated that GCP showed better effects through the gut microbiota [10], total DNA was extracted from the Normal group, Model group, and GCP‐treated group, fecal samples for further 16S rRNA sequencing to investigate gut microbiota. The Normal group, Model group, and GCP‐treated group displayed 4002, 4993, and 4819 unique OTUs, respectively, which indicated that orally administered GCP restored the number of OTUs to some extent (Fig. 5A). The rarefaction curves and Shannon curves were generated from OTUs, with 97% identity achieved in all samples (Fig. 5B,C). This indicated that the testing samples were sufficient and the amount of data was reasonable for the investigation of fecal microbiota. Microbial community diversity was described by measuring Shannon indices. The data showed that Shannon indices in the Model group were much higher than those in the GCP‐treated group and the Normal group (Fig. 5D). Additionally, we investigated the overall microbial community structure similarity by PCoA analysis unweighted. The community composition structure and aggregation similarity of the GCP‐treated group were different from the Model group, but the same in the Normal group (Fig. 5E).

To further understand the specific changes in the microbial community, we analyzed the taxonomic composition of each group sample. At the phylum level, the microbiota were mainly composed of *Bacteroidetes*, *Firmicutes*, *Epsilonbacteraeota*, *Proteobacteria*, and *Tenericutes*, among which *Bacteroidetes* and *Firmicutes* accounted for more than 80% of them. As we can see in Fig. 5F, the abundance of *Bacteroidetes* and *Tenericutes* was increased and the abundance of *Firmicutes* and *Proteobacteria* decreased in the Model group. In contrast, GCP reversed these changes, *Bacteroidetes*, as well as *Tenericutes*, downregulated and *Proteobacteria*, as well as *Firmicutes*, upregulated. Additionally, the abundance of *Verrucomicrobia* was increased and the abundance of *Epsilonbacteraeota* was decreased in the GCP‐treated group. At the genus level, *Alistipes*, *Uncultured Bacteroidales bacterium*, *Lachnospiraceae. NK4A136.group*, *Ruminococcaceae UCG.014*, *Parabacteroides*, *Alloprevotella*, *Akkermansia*, *Lactobacillus*, and *Bacteroides* were the eight most predominant microbiota. The results showed an abundance of *Bacteroides*, *Alloprevotrlla*, and *Lachnospiraceae. NK4A136* greatly raised and *Lactobacillus* decreased in the Model group. However, GCP treatment could weaken the influences, the abundance of *Bacteroides*, *Alloprevotrlla*, *Lachnospiraceae*, and *NK4A136* reduced, and *Lactobacillus* recovered. We also found that *Parabacteroides* and *Akkermansia* are greatly increased in the GCP‐treated group compared with the Normal and Model groups (Fig. 5G).

### GCP regulated local cytokines in gut mucosa via gut microbiota and gut mucosa TLRs/NF‐κB pathway in tumor‐bearing mice

**Fig. 6:**
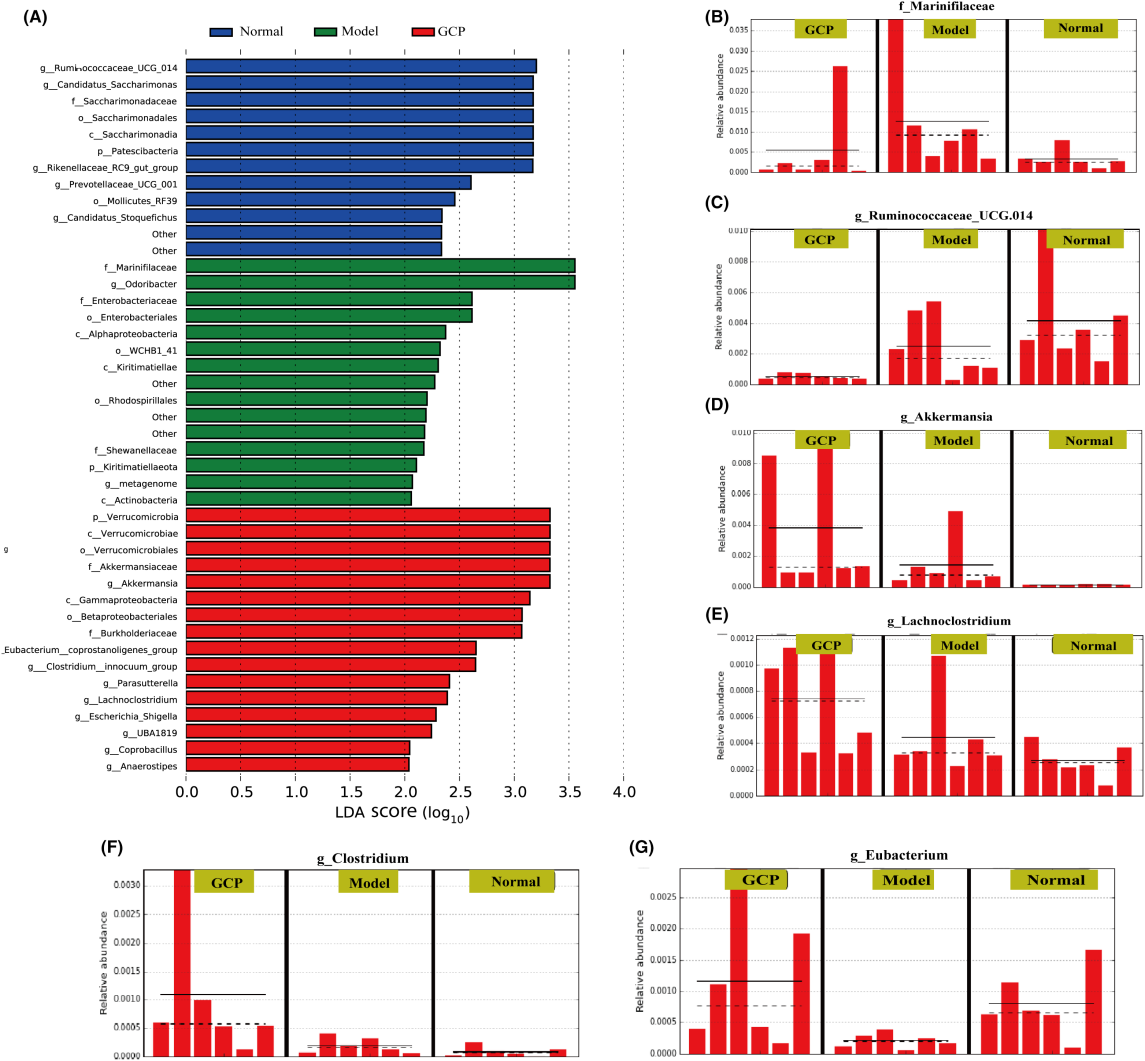
GCP significantly activated the expression of TLRs/NK‐κB pathway. (A) Cladograms generated by LEfSe indicate differences in the bacterial taxa in Normal, Model, and GCP groups. Red bars indicate taxa with enrichment in the GCP group, green bars indicate taxa with enrichment in the Model group, and blue bars indicate taxa with enrichment in the Normal group. (B–G) The abundance of six potential diagnostic gut microbiota biomarkers in each sample including *f_Marinifilaceae*, *g_Ruminococcaceae_UCG.014*, *g_Akkermansia*, *g_Lachnoclostridium*, *g_Clostridium*, *g_Eubacterium*.

**Fig. 7:**
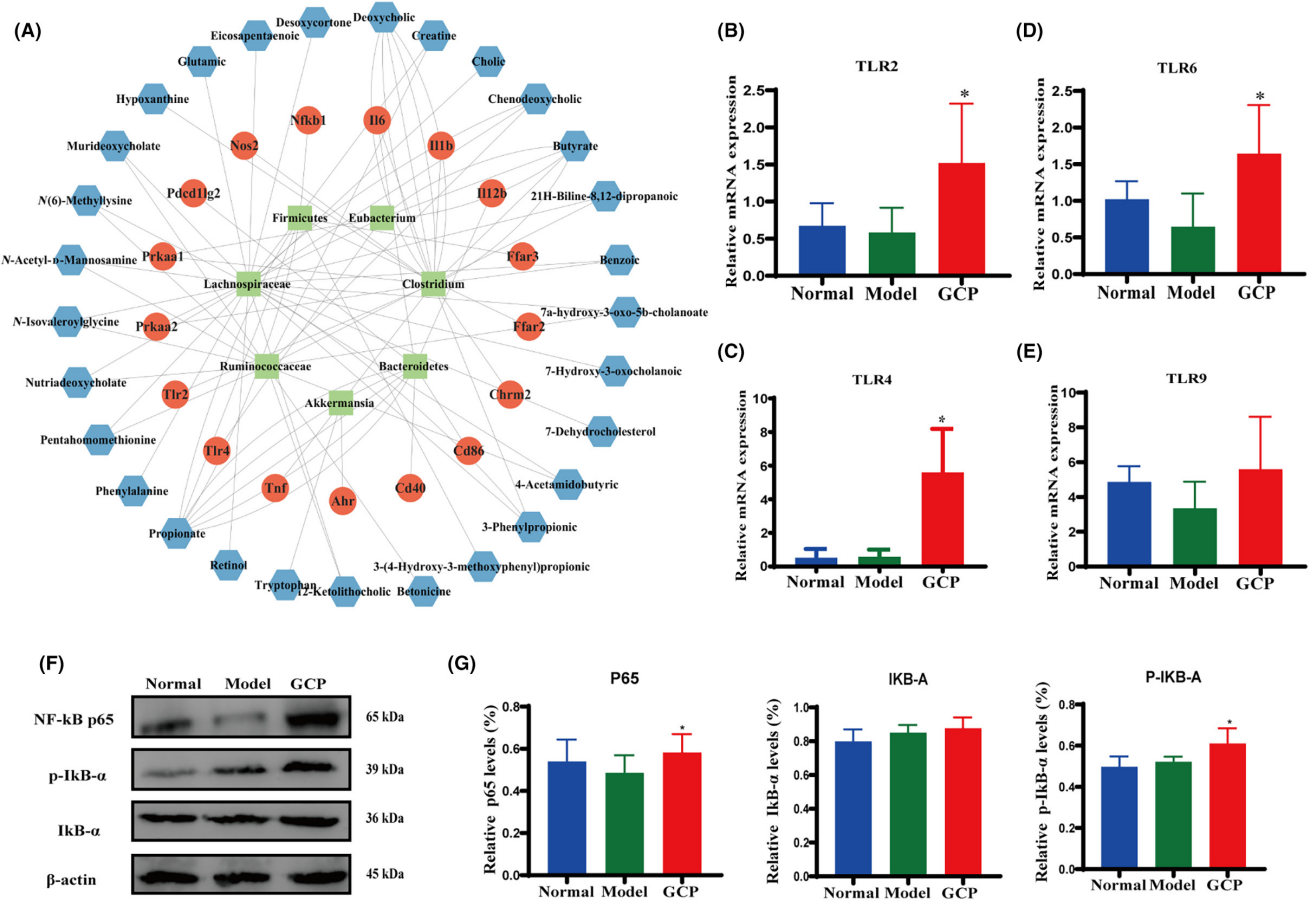
GCP significantly activated the expression of the TLRs/NK‐κB pathway. (A) Visual analysis of symbiotic networks for potential diagnostic gut microbiota biomarkers in three groups stands for positive interactions between nodes, and green links stand for negative interactions. (B–E) RT‐qPCR was used to detect the expression of TLR2, TLR4, TLR6, and TLR9 genes in intestinal epithelial (*P < 0.05, *n* = 6 Model :vs. GCP). (F) Representative western blotting images of p65, p‐IκB‐α, IκB‐α protein in intestinal epithelial. (G) Western blotting statistical results for p65, p‐IκB‐α, IκB‐α proteins in the intestinal epithelial (*P < 0.05, *n* = 6 Model :vs. GCP).

To find the key target genes of GCP based on gut microbiota, we performed LEfSe analysis among the Normal group, Model group, and GCP group. LDA results showed 12 discriminative features in the normal group (LDA > 2.0) (Fig. 6A). The Model group showed 15 dominant microorganisms and the GCP‐treated group showed 16 dominant microorganisms as well (LDA > 2.0). Then, concerning the results of LEfSe analysis of different gut microbiota, we finally selected five potential diagnostic gut microbiota biomarkers which are *f_Marinifilaceae*, *g_Ruminococcaceae_UCG.014*, *g_Akkermansia*, *g_Lachnoclostridium*, *g_Clostridium*, and *g_Eubacterium*. The relative abundances of these six bacterial genera in each group are shown in Fig. 6B–G. Finally, we used the gutMgene database to find the gut microbiota that changed after GCP treatment. Target genes of gut microbiota that changed after GCP treatment were depicted using cystoscope (version 3.9.1). The results showed that changed gut microbiota was associated with 29 metabolites and could target 17 genes, including NF‐κB, TLR2, and TLR4 (Fig. 7A).

As shown in Fig. 7B–D, GCP‐treated resulted in a remarkable increase of TLR2, TLR4, and TLR6 mRNA expression (*P* < 0.05). However, the TLR9 mRNA expression displayed no significant difference in the three groups (Fig. 7E) (*P* > 0.05). Furthermore, the protein expression levels of p‐IκB‐α and NF‐κB p65 were significantly increased after GCP administration (Fig. 7F,G) (*P* < 0.05).

**Fig. 8:**
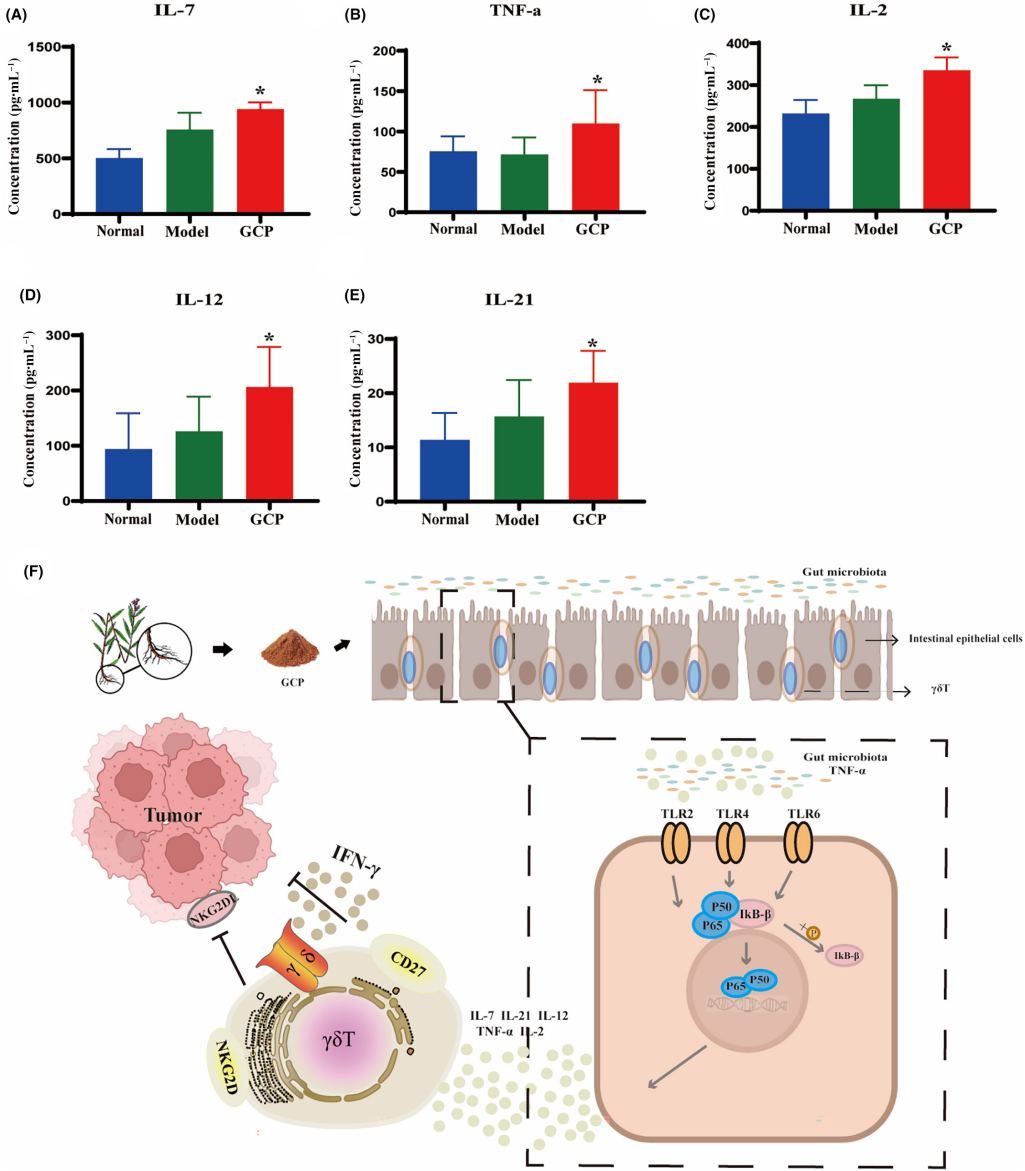
GCP significantly promoted the secretion of mucosal cytokines. (A–E) ELISA was used to detect cytokine IL‐7, TNF‐α, IL‐2, IL‐12, and IL‐21 concentration in intestinal mucosa (*P < 0.05, *n* = 6 Model :vs. GCP). (F) Antitumor immunity effect of GCP on intestinal epithelial γδT cells through gut microbiota and the TLRs/NF‐κB signaling pathway.

It is well known that the fate of γδT cells, including proliferation, maintenance, differentiation, and activation, is closely related to a variety of cytokines. Here we detected five major cytokines, IL‐2, IL‐7, IL‐12, IL‐21, and TNF‐α, which are involved in intestinal γδT cell maintenance and activation. Compared with the Normal group and Model group, after GCP treatment the concentrations of IL‐7, TNF‐α, IL‐2, IL‐12, and IL‐21 were significantly enhanced than those in the Model group (*P* < 0.05) (Fig. 8A–E), suggesting that GCP influenced the local cytokines‐milieu of the gut mucosa and regulate γδT cells differentiation.

## Discussion

*Glycyrrhiza uralensis* is a kind of traditional Chinese medicine with a long history of being used in medical treatment [28]. Multiple biofunctions have been found, such as immunomodulatory, antihyperglycemic, antiinflammatory, antioxidant, and antiviral activities [29, 30]. Glycyrrhiza polysaccharides (GCP) are one of the primary active ingredients of *G. uralensis*. Modern pharmacological evidence shows that GCP has strong antitumor activity and enhances host immune function. An optimized method of extraction, separation, and purification of GCP has been established in our laboratory [14]. Our previous studies have found that GCP can exert an antitumor effect by increasing the expression of the serum factor IL‐7 in both serum and intestinal mucosa [16, 17], which is consistent with the results found in this study. Our research suggested that GCP treatment has antitumor effects in mice and may improve the quality of life of tumor‐bearing mice. The immunophenotypic analysis further suggested that GCP can inhibit tumor growth by inhibiting cell proliferation. and modulate systemic immunity indirectly. We found that GCP can effectively increase the spleen index and increase the proportion of CD3^+^T cells in the spleen and tumor tissue of mice. In addition, the levels of serum cytokines TNF‐α, IFN‐γ, IL‐2, IL‐12, and IL‐7 significantly increased. These results suggest that the antitumor effect of GCP is related to the activation of tumor immune response in tumor‐bearing mice.

On the other hand, the gut mucosal immune system serves as the starting point for oral administration of polysaccharides to exert immune efficacy and regulate human immune function [31, 32]. The area of the gut mucosa alone reaches about 400 square meters due to the existence of structures such as folds, villi, and microvilli. Therefore, the gut mucosa is the largest area of the immune response area and the primary portal for the human body to interact with the external environment [33]. As the main component of gut mucosal immunity, gut mucosal immune cells, including T cells, dendritic cells, B cells, and macrophages, play an important role in maintaining intestinal mucosal homeostasis [34, 35]. Studies have shown that polysaccharides can significantly regulate gut mucosal γδT cells to enhance immunity. For example, Lentinan can increase the proportion of intestinal mucosal γδT cells and improve the life quality of immunosuppressed mice [36]. Astragalus polysaccharides can partly promote the secretion of IFN‐γ and TNF‐α by intestinal mucosal γδT cells, and inhibit the growth of tumors in tumor‐bearing mice [37]. Our research revealed that GCP greatly increased the proportion of γδT cells in the intestinal mucosa and showed antitumor activity. Recent research shows that gut γδT cells, which are IFN‐γ‐secreting, but not IL‐17–secreting, are glycolytic and exert antitumor activity, and analysis of human samples also pointed to glycolytic γδT cells that were preferentially found in the tumor‐adjacent areas [38]. γδT cells can recognize the tumor cells by expressing NKG2D and secrete the IFN‐γ to inhibit the tumor growth [39]. Meanwhile, studies have found that CD27^+^γδT cells secrete IFN‐γ to inhibit tumor cell growth, while CD27^−^γδT cells produce IL‐17 [40]. Based on the above evidence, our research found that GCP‐treatment enhanced the expression of NKG2D and the differentiation phenotype CD27^+^ of the gut mucosal γδT cells, and the secretion of the cytokine IFN‐γ. Indubitably, the proliferation and differentiation of γδT cells can be affected by many factors, among which cytokines are crucial. Studies have found that TNF‐α and IL‐21 can increase the proportion of γδT cells that secrete IFN‐γ in *in vivo* experiments [41]. IL‐2 is a cytokine that maintains the growth of T cells. Recent studies have shown that IL‐2 increases the expression of NKG2D in γδT cells *in vitro* [42]. Moreover, IL‐12 and IL‐18 increase the secretion of IFN‐γ in human γδT cells and promote tumor cell apoptosis [43]. IL‐7 and IL‐15 are key regulators of CD27 expression on the surface of γδT cells [44]. Meanwhile, our study found that GCP increased the secretion of intestinal mucosal cytokines IL‐2, IL‐7, IL‐12, IL‐21, and TNF‐α. These results indicate that GCP could promote the proliferation and differentiation of γδT cells toward IFN‐γ by stimulating intestinal mucosal cytokine secretion. Importantly, our research found that GCP greatly increased the proportion of γδT cells in the spleen and tumor, and the infiltrating γδT cells in the tumor were CD27^+^ type. The exciting finding is that GCP improved the immunity of gut mucosal γδT, while significantly activating overall immunity and tumor local immunity.

TLRs are a type of pattern recognition receptors that play critical roles in the innate immune system and exist in different cell types, including epithelial cells and immune cells. Activation of TLRs is closely related to gut microbiota. Probiotic *Clostridium butyricum* promotes IL‐10 production by CD11b^+^CD11c(int) intermediate F4/80^+^ intestinal macrophages via the TLR2/MyD88 signaling pathway in dextran sodium sulfate (DSS)‐induced colitis [45]. TLR4 senses bacterial components such as lipopolysaccharide, and thus induces signal transduction, contributing to the production of cytokines, antimicrobial molecules, and mucus by IECs [46]. Reviews have shown that as potential prebiotics for utilizing gut microbiota, polysaccharides can result in a change in gut microbial diversity and composition [47]. Cultured *C. sinensis* polysaccharides (CSP) significantly change the diversity of the intestinal community, restore Cy‐induced intestinal mucosal immune imbalance, and upregulate the mRNA expression of TLR2, TLR4, and TLR6 [23]. Our team conducted microbiota depletion and fecal microbiota transplantation (FMT) experiments in the early study and found that the antitumor effect of GCP depends on the presence of gut microbiota, but the specific mechanism of action is not clear [10]. Our research in this article showed that the Model group had an increased abundance of *Bacteroides* and *Tenerella* and a decreased abundance of *Firmicutes* and *Proteus*. In contrast, GCP reversed these changes, with the downregulation of *Bacteroides* and *Tenericutes* and the upregulation of *Proteobacteria* and *Firmicutes*. The gutMgene database covers the links between 273 gut microbes in mice and 33 diseases and 151 interventions, all validated by 16S amplicon sequencing, metagenomic sequencing, and qPCR [48]. After database screening and visual analysis, we finally observed that the gut microbiota regulated by GCP is mainly related to metabolites of 29 metabolites such as butyrate, creatine, benzoic, glutamic, and related to 17 genes including TLR4, TLR2, NF‐κB, and TNF. Additionally, together with secretory proteins MD‐2 and CD14, TLRs stimulate downstream signaling cascades that result in activation of NF‐κB and induction of cytokine secretion [49, 50]. For one thing, NF‐κB is a ubiquitous transcription factor and plays a role in the regulation of gene expression related to many cell processes. It can regulate the expression of cytokines and serve as a key response to environmental changes. When NF‐κB is activated, the inhibitory NF‐κB protein IκBs will be phosphorylated by IKK, thus transferring the activated NF‐κB to the nucleus. After the NF‐κB pathway is activated, it can promote the secretion of cytokines such as TNF‐α, and at the same time, TNF‐α can continue to activate the NF‐κB pathway to form a circulation pathway and regulate the intestinal mucosal microenvironment [51]. For another, TLRs agonists have been widely used in the clinical treatment of malignant tumors [52]. The administration of TLRs agonists can induce the immune response of CD8 T cells to exert a tumor‐suppressing effect [53]. Our data indicated that GCP increased the mRNA levels of TLR‐2, TLR‐4, and TLR‐6, and the protein levels of p65 and p‐IκB‐α in GCP‐intervened intestinal epithelial cells. This indicated that GCP stimulated the secretion of cytokines via the TLRs/NF‐κB pathway and affected the differentiation of γδT cells. This might provide a new treatment strategy for issues like compromised immunity and immunosuppression caused by traditional radiotherapy and chemotherapy drugs. In order to promote the application of GCP in clinical treatment, its pharmacokinetics and toxicity should be further studied. In addition, there are still several missing links to be elucidated in future studies, including the exploration of the mechanisms by which GCP remotely regulates tumor immunity and systemic immunity.

## Conclusion

Our research demonstrated that gut epithelia γδT cells are one of the main contributors to the antitumor effect of GCP. GCP promotes the differentiation of gut epithelia γδT cells into the IFN‐γ‐producing subtype through regulated local cytokines in the gut mucosa. GCP regulated local cytokines in gut mucosa via gut microbiota and the gut mucosa TLRs/NF‐κB pathway (Fig. 8F). It further indicated that natural medicine and its active ingredients may have a potential value in tumor immunotherapy with γδT cells. Further studies, including clinical trials, are required to validate the results presented here.

## Conflict of interest

The authors declare no conflicts of interest.

### Peer review

The peer review history for this article is available at https://www.webofscience.com/api/gateway/wos/peer‐review/10.1002/2211‐5463.13800.

## Author contributions

Thanks to the conceptualization and designed study of SZ, XW, and YB, the experiment and original draft preparation of YC, LB, and ZL, and the data curation of ZL, LB, YP, PX, and XS. All authors have read and approved the final article.

## Supporting information


**Fig. S1.** GCP inhibited the development of tumors through an increase in the percentage of infiltration γδT cells in the tumors.

## Data Availability

The datasets used and/or analyzed during the current study are available from the corresponding author on reasonable request.
