# Supplementary material for: Glycyrrhiza polysaccharides may have an antitumor effect in γδT cells through gut microbiota and TLRs/NF‐κB pathway in mice
Source: FEBS Open Bio. 2024 Apr 11;14(6):1011–27. doi: 10.1002/2211-5463.13800 (PMC11148121; doi:10.1002/2211-5463.13800)
Supplement: Supplementary file 1 — Fig. S1. GCP inhibited the development of tumors through an increase in the percentage of infiltration γδT cells in the tumors. [file FEB4-14-1011-s001.docx]

**Supporting Information**

**
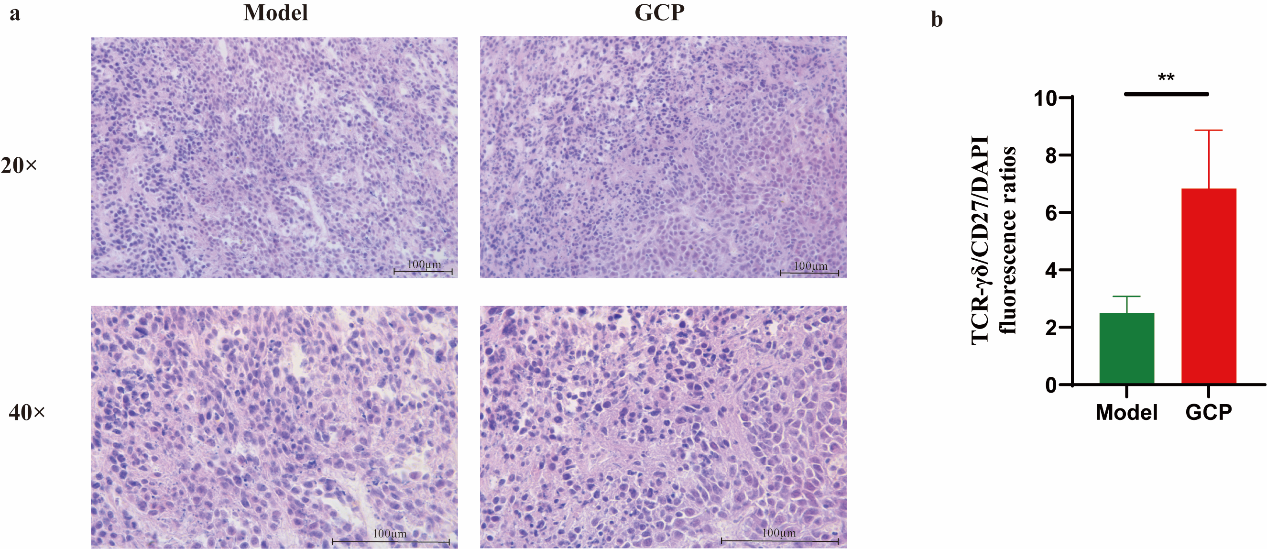
Fig S1** GCP inhibited the development of tumors through increase the percentage of infiltration γδT cells in the tumors (a) Representative images of HE staining of tumors. (Scale bar, 100µm) (b) Immunofluorescence staining statistical results for TCR-γδ and CD27 in tumors. (**P*<0.01 (n=3)
